# Blood component therapy for dry eye disease: a systematic review and network meta-analysis

**DOI:** 10.3389/fmed.2024.1500160

**Published:** 2024-12-16

**Authors:** Yu Zhang, Ning Li, Ziying Ge, Fang Li

**Affiliations:** Department of Ophthalmology, Zhangye People’s Hospital Affiliated to Hexi University, Zhangye, China

**Keywords:** dry eye disease, blood component therapy, systematic review, network meta-analysis, randomized controlled trial

## Abstract

**Objective:**

Blood component therapy has shown promising potential as an emerging treatment for dry eye disease; however, it remains unclear which specific blood component is the most effective. This study aims to compare the efficacy of different blood components in the treatment of dry eye disease through a network meta-analysis, with the goal of providing the latest and most reliable evidence for clinical practice.

**Methods:**

We conducted a systematic search of the PubMed, Web of Science, Cochrane, Embase, and Scopus databases, with the search concluding on June 1, 2024. Two independent researchers performed literature screening, data extraction, and quality assessment.

**Results:**

A total of 16 randomized controlled trials (RCTs) involving 898 patients with dry eye disease were included. Six different blood components were utilized in treating dry eye disease, with platelet-rich plasma (PRP) being the most widely used. The results of the network meta-analysis indicated that platelet-rich plasma eye drops (PRPD) significantly outperformed artificial tears (AT) in improving the corneal fluorescein staining score (CFSS), while autologous serum (ALS) and umbilical cord serum (UCS) also demonstrated significantly better effects than AT in enhancing tear break-up time (TBUT). Additionally, ALS, PRP injection (PRPI), and PRPD showed significantly superior outcomes compared to AT in improving the ocular surface disease index (OSDI). However, no statistically significant differences were found among the various treatment modalities regarding their effects on Schirmer’s I value, CFSS, TBUT, and OSDI. SUCRA analysis predicted that UCS was the most effective in improving Schirmer’s I value and TBUT, while PRP excelled in enhancing CFSS and OSDI. Limitations such as publication bias and issues related to randomization, allocation concealment, and blinding may affect the reliability of the current findings.

**Conclusion:**

Blood component therapy can significantly improve the pathological damage and ocular surface health in patients with dry eye disease. For those with aqueous-deficient dry eye, UCS may represent the optimal treatment option. In contrast, for patients with more severe corneal epithelial damage, PRP may offer a more effective therapeutic approach.

**Systematic Review Registration:**

https://www.crd.york.ac.uk/PROSPERO/, CRD42024534091.

## 1 Background

Dry eye disease is a common and complex ocular surface dysfunction characterized by a wide-ranging prevalence globally, estimated to be between 5 and 50%, with a significant increase in incidence observed with advancing age (1–3). Its symptoms are diverse, including dryness, itching, photophobia, foreign body sensation, burning, excessive tearing, and blurred vision. In severe cases, it can lead to corneal ulcers or vision loss. Furthermore, prolonged ocular discomfort may result in anxiety, depression, and sleep disturbances, significantly impacting the economic, social, and psychological wellbeing of patients (3–6). Currently, artificial tears are the primary treatment for dry eye disease, alleviating symptoms by increasing ocular surface moisture, reducing tear osmolarity, and decreasing soluble inflammatory mediators (1, 7). However, the effects of artificial tears are often transient, making it challenging to maintain long-term control of chronic inflammatory responses on the ocular surface. Additionally, preservatives found in many artificial tear products can irritate the ocular surface and even exacerbate symptoms, limiting their feasibility for long-term use (8, 9). Therefore, there is an urgent need to develop more durable and effective treatment options.

In recent years, blood component-based eye drop therapies have garnered attention, particularly for their potential clinical advantages in managing dry eye disease and other ocular surface disorders (10). Blood-derived products such as autologous serum and platelet-rich plasma are rich in various growth factors, anti-inflammatory agents, and nutrients, playing a unique role in promoting ocular surface cell repair, reducing inflammatory responses, and stabilizing the tear film. These therapies not only exhibit high biocompatibility but also allow for personalized treatment tailored to the specific conditions of patients, making them suitable for various types of dry eye disease and gradually establishing themselves as a promising treatment option. Nevertheless, existing evidence has certain limitations. First, the published studies included data from autologous pre- and post-comparative studies or observational research, rather than randomized controlled trials, which raises concerns about the reliability of the results (11). Second, some studies utilized endpoint follow-up values for data analysis instead of the changes in values before and after treatment. They acknowledged that differences in baseline characteristics among patients could lead to variations in endpoint values, thereby affecting the reliability of the meta-analysis results (12). Additionally, research by Quan et al. (13) indicated slight improvements in certain metrics (such as staining scores) with autologous serum, but overall efficacy did not demonstrate significant superiority.

Currently published meta-analyses exhibit deficiencies in data analysis methods and evidence quality, particularly regarding the efficacy assessment of blood component therapies for dry eye disease, where considerable controversy remains. Furthermore, traditional meta-analyses often limit comparisons to two treatment modalities, while existing clinical studies frequently compare a single blood component with other control treatments, making systematic evaluation of the relative effects of multiple blood components in treating dry eye disease highly challenging. Simultaneously, we conducted a search in the PubMed and Web of Science databases using the search terms (xerophthalmia OR dry eye) AND network meta-analysis. To date, we found no published network meta-analysis specifically addressing the treatment of dry eye disease with different blood components. In light of these shortcomings and challenges, this study aims to systematically evaluate the relative efficacy of various blood components in the treatment of dry eye disease through a network meta-analysis, with the goal of providing more comprehensive and reliable evidence to support clinical decision-making.

## 2 Materials and methods

This study adheres to the Preferred Reporting Items for Systematic Reviews and Meta-Analyses (PRISMA) guidelines (14), and has been registered in the PROSPERO database (CRD42024534091). Detailed inclusion and exclusion criteria were established based on the PICOs framework.

### 2.1 Inclusion and exclusion criteria

Patients (P): patients who meet the clinical diagnostic criteria for dry eye disease will be included, with no restrictions on age, gender, source of cases, or duration of the condition.

Interventions (I): interventions will include blood component therapies such as serum, platelets, plasma, and whole blood, with a treatment duration exceeding 2 weeks.

Control (C): control groups will consist of artificial tears or different blood components from those used in the experimental group.

Outcome (O): the primary outcomes will include Schirmer’s I value, corneal fluorescein staining score (CFSS), tear break-up time (TBUT), and ocular surface disease index (OSDI).

Type of study (S): only RCTs investigating blood component therapies for dry eye disease will be included, regardless of whether allocation concealment and blinding were employed.

Exclusion criteria: (1) Studies that do not involve blood component therapies for dry eye disease or that focus on short-term treatments (≤2 weeks) (15) will be excluded. (2) Reviews, letters, comments, case reports, animal or laboratory studies, and conference abstracts will be excluded. (3) Studies that do not report the predefined outcome measures will be excluded. (4) Studies for which full text or data cannot be obtained will be excluded.

### 2.2 Data sources and searches

A comprehensive search was conducted across the PubMed, Web of Science, Cochrane, Embase, and Scopus databases, covering the period from the inception of each database until June 1, 2024. The search strategy combined both subject headings and free-text terms, with keywords including: (blood OR hematological OR serum OR blood serum OR hemocyte OR blood cell OR blood cells OR thrombocyte OR platelet OR platelets OR plasma OR plasmatic OR erythrocyte OR erythrocytes OR red blood cell OR erythrocytic OR leucocyte OR white blood cell OR leukocyte OR leukocytes) AND (xerophthalmia OR dry eye OR dry eye syndrome OR corneal and conjunctival xerosis). Additionally, relevant literature was manually searched by reviewing the references of identified studies to ensure no pertinent research was overlooked. The specific search strategy is detailed in Supplementary Table 1.

### 2.3 Literature screening, data extraction, and quality assessment

All search results were imported into EndNote 9.1 reference management software for organization. After removing duplicates, two experienced systematic review researchers independently screened the literature based on the inclusion and exclusion criteria, followed by cross-checking to ensure consistency and accuracy in the selection process. The literature screening was divided into two phases: initial screening and secondary screening. During the initial screening phase, studies that clearly did not meet the inclusion criteria were excluded by reviewing the titles and abstracts. In the secondary screening phase, the full texts of the remaining studies were carefully read to further confirm whether they met all inclusion criteria, with non-compliant studies being excluded. In cases of disagreement, the two researchers discussed the issues to reach a resolution, and a third-party researcher was consulted if necessary.

Data were extracted using a pre-established data extraction form, which included: (1) Basic information: authors, publication year, country, study type, severity, sample size, age, gender, treatment method, frequency, and duration of treatment, (2) Predefined outcome measures: Schirmer’s I value, CFSC, TBUT, and OSDI, (3) Information related to bias risk assessment.

This study employed the Cochrane Risk of Bias Assessment Tool to systematically evaluate the included RCTs (16). The assessment process comprised five main domains: selection bias, performance bias, detection bias, attrition bias, and other biases. Each domain was evaluated based on criteria for low risk, high risk, and unclear risk, culminating in an overall assessment of the risk of bias. Data analysis was conducted using RevMan software for data entry and visual representation, facilitating an intuitive understanding of the assessment results. Through this approach, we were able to effectively identify bias risks in the RCTs, thereby enhancing the credibility of the meta-analysis results.

### 2.4 Statistical analysis

Based on a frequentist approach, we conducted a network meta-analysis using Stata 16.0 software, with the effect size measured as the standardized mean difference (SMD) and calculating a 95% confidence interval (CI). A difference was considered statistically significant if the 95% CI did not include zero. First, we used the Network package to create a network evidence plot, where each node represents a different blood component treatment regimen. The size of each node indicates the sample size for that treatment, while the thickness of the lines connecting the nodes reflect the number of studies included. To compare the effectiveness of different blood components, we employed the surface under the cumulative ranking curve (SUCRA) method; a higher SUCRA value indicates better treatment efficacy for that blood component. The choice of SUCRA ranking is due to its ability to comprehensively consider the relative effects of all interventions and provide a more intuitive ranking result. Compared to other ranking methods, SUCRA offers greater statistical efficiency and reliability when handling multiple comparisons. Finally, we generated a funnel plot to assess the presence of small sample effects and publication bias. All statistical analyses were deemed statistically significant at *p* < 0.05.

## 3 Results

### 3.1 Overview of studies

**Figure 1:**
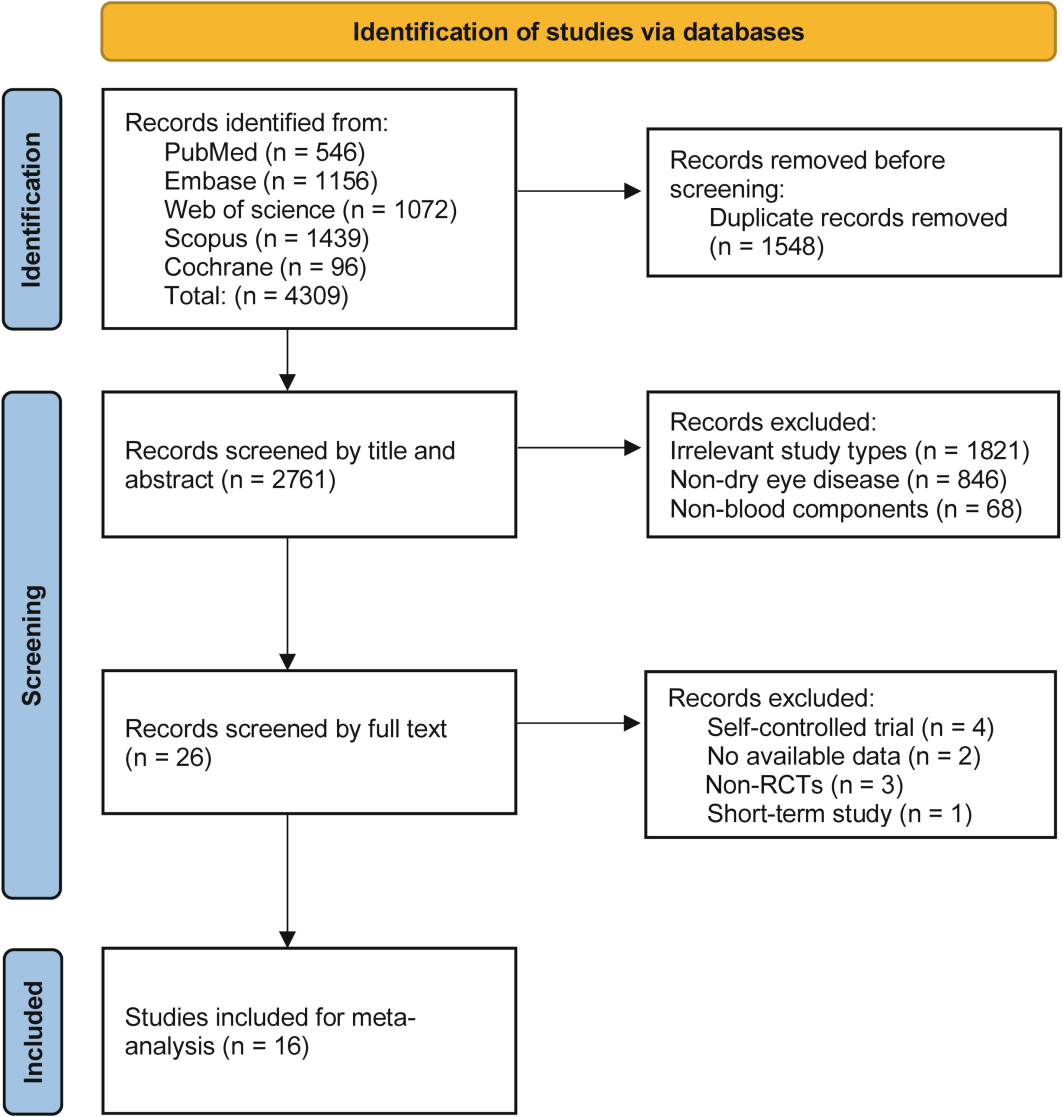
PRISMA flow diagram.

A total of 4,309 relevant articles were identified through preliminary searches. After screening, 1,548 duplicate articles and 2,745 that did not meet the inclusion criteria were removed, resulting in the inclusion of 16 RCTs (17–32). The literature screening process is detailed in Figure 1. The included studies involved a total of 898 patients with dry eye disease, comprising 179 males and 528 females, while three studies did not report gender distribution. The sample sizes ranged from 20 to 144 participants. Except for four studies that did not report the severity of dry eye disease, the remaining studies recruited patients with moderate to severe dry eye. All patients were over 20 years of age, with one study not providing age data.

In this study, six blood components were utilized for the treatment of dry eye disease, including platelet-rich plasma (PRP), umbilical cord serum (UCS), allogeneic peripheral blood serum (APS), calf blood deproteinized extract eye drops (CBDE), autologous whole blood (AWB), and autologous serum (ALS). Among these, PRP was the most widely used, with two studies employing platelet-rich plasma injections (PRPI) and four studies using platelet-rich plasma eye drops (PRPD). Treatment frequency across studies ranged from four to six times per day, with treatment durations of more than 3 weeks. Detailed information on the included studies can be found in Supplementary Table 2.

### 3.2 Risk of Bias assessment results

**Figure 2:**
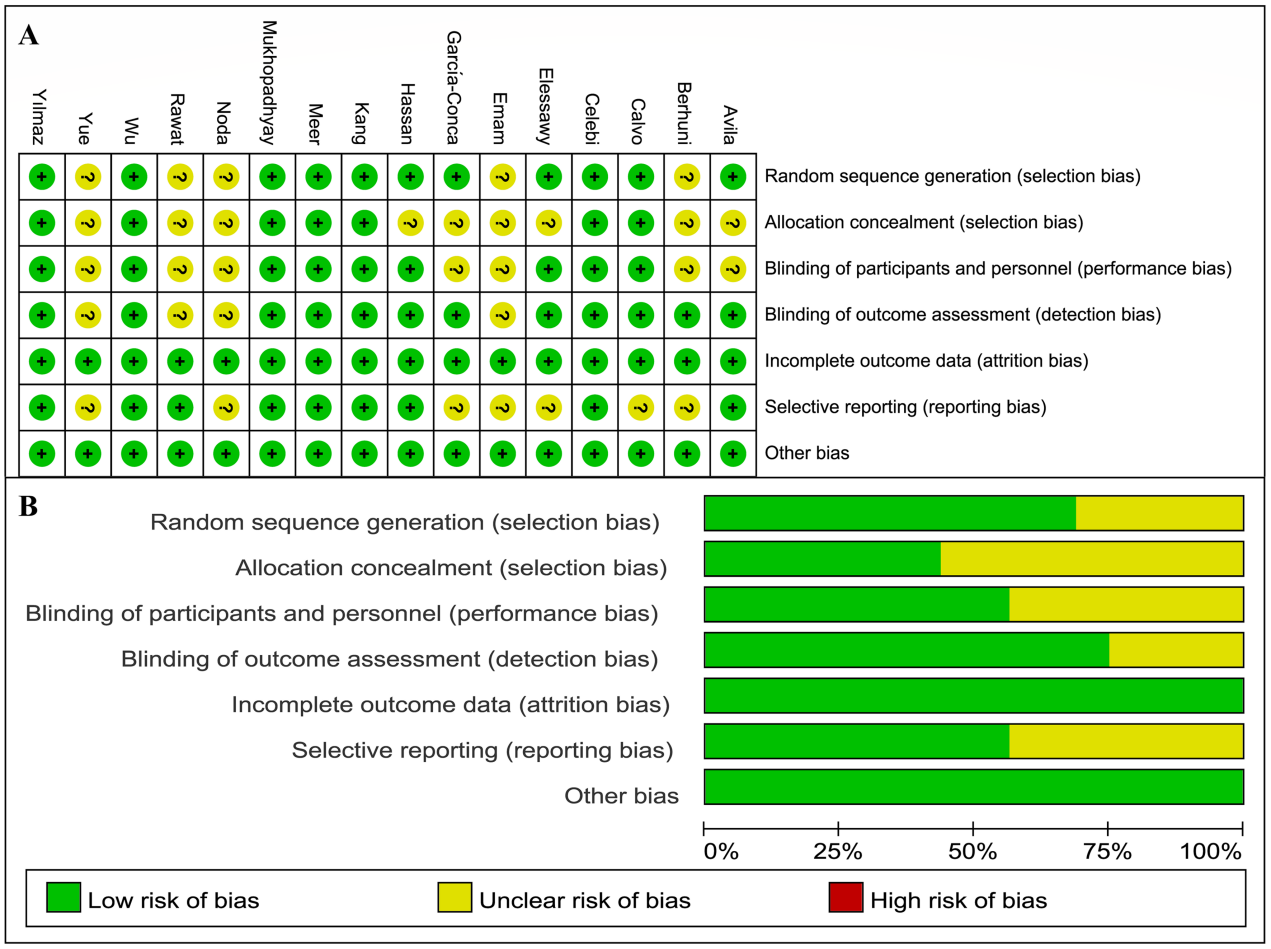
Risk of bias assessment results. **(A)** Risk of bias assessment results for individual studies. **(B)** Risk of bias assessment results for each item.

The risk of bias was assessed for the 16 studies, revealing an overall low risk. Eleven studies employed computer randomization or random number tables for group allocation, while five studies did not clearly report their randomization methods. Regarding allocation concealment, seven studies used sealed envelopes for allocation, while the remaining nine did not provide relevant information. Nine studies implemented blinding for both researchers and patients, and 12 studies blinded the outcome assessors. The loss to follow-up rate was maintained at 20% or less, or no losses occurred in any of the studies. In terms of selective reporting, nine studies were registered on clinical trial platforms and provided complete study protocols. Additionally, all studies declared no potential conflicts of interest. Details are presented in Figure 2.

### 3.3 Evidence network

**Figure 3:**
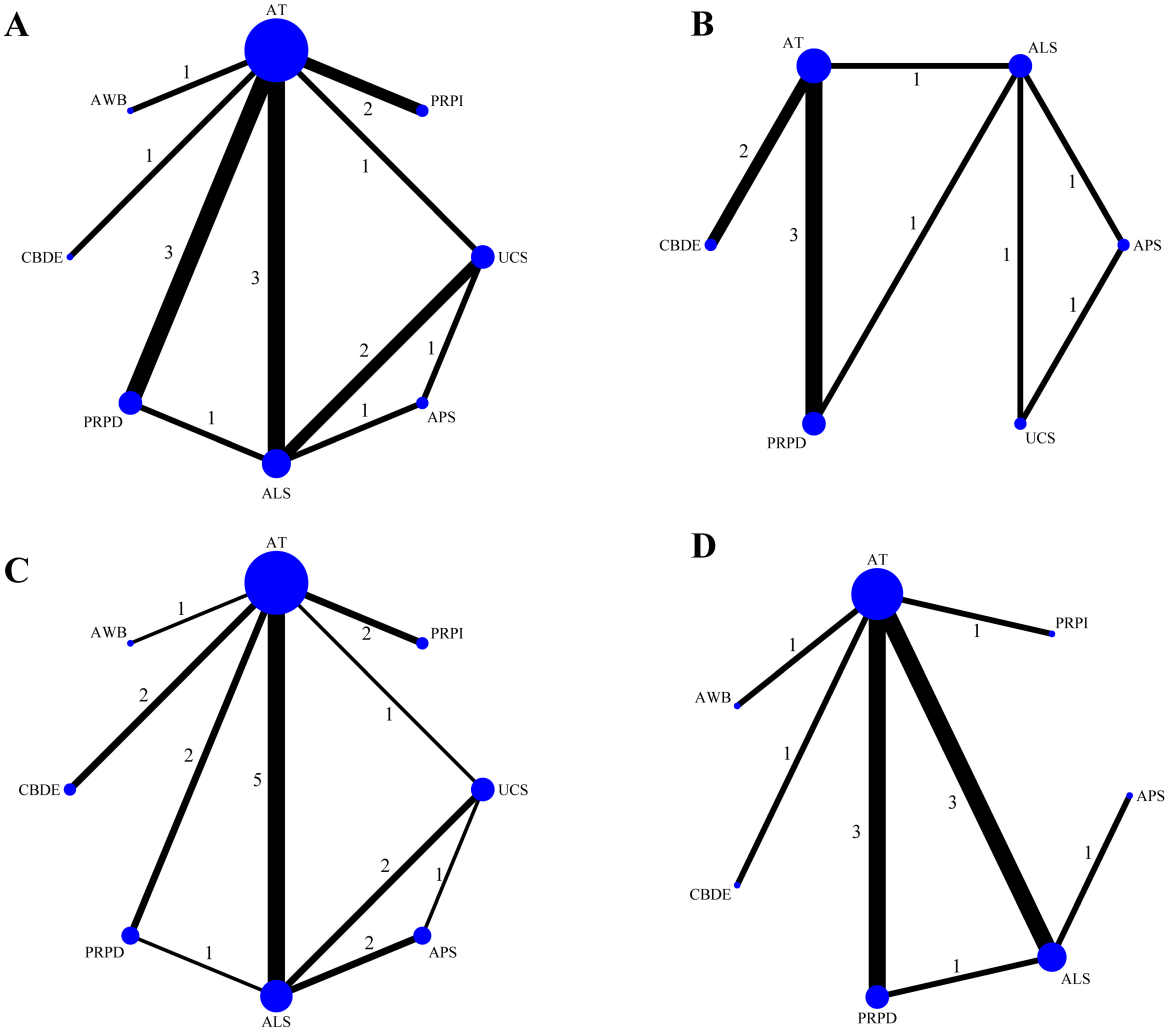
Network evidence plot. **(A)** Schirmer’s I value; **(B)** CFSS; **(C)** TBUT; **(D)** OSDI. The size of the points represents the total sample size, while the thickness of the lines indicates the number of studies comparing different blood components directly.

Among the 16 included studies, 12 reported Schirmer’s I value, involving eight treatment modalities, with ALS as the most commonly used blood component (Figure 3A). Eight studies reported CFSS, involving six treatment modalities, with ALS again being the most frequently used treatment (Figure 3B). Fifteen studies reported TBUT, involving eight treatment methods, with ALS being the most commonly utilized (Figure 3C). Eleven studies provided OSDI data, involving seven treatment modalities, with ALS remaining the most prevalent treatment component (Figure 3D). Figure 3 illustrates direct comparisons between different treatment modalities.

### 3.4 Network meta-analysis

**Figure 4:**
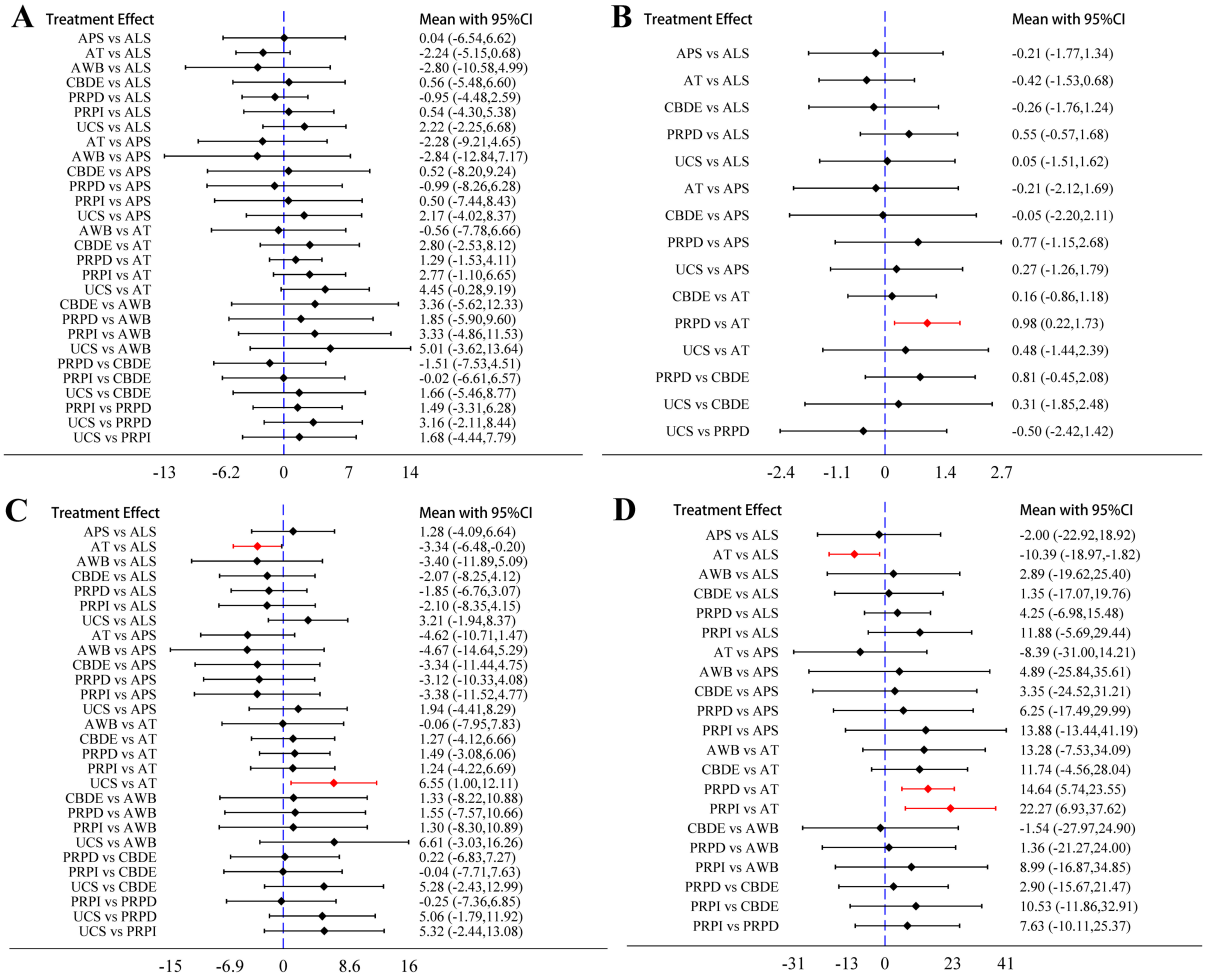
Network meta-analysis results. **(A)** Schirmer’s I value; **(B)** CFSS; **(C)** TBUT; **(D)** OSDI. The diamonds and horizontal lines in the forest plots represent the effect sizes and their 95% confidence intervals, with the blue dashed line indicating the null effect line. When the horizontal lines intersect the null effect line, it indicates no significant difference in treatment effects between the two blood components; conversely, if they do not intersect, it suggests a statistically significant difference. The sections highlighted in red indicate results with statistical significance.

The analysis based on Schirmer’s I value indicated no statistically significant differences in the improvement of lacrimal gland function among the different blood components (Figure 4A). The analysis based on CFSS revealed that PRPD had a significantly better treatment effect compared to artificial tears (AT); however, no significant statistical differences were observed among the other blood components (Figure 4B). The analysis based on TBUT showed that ALS and UCS had significantly better treatment effects compared to AT, while no significant statistical differences were found among the other blood components (Figure 4C). The analysis based on OSDI indicated that ALS, PRPI, and PRPD had significantly better treatment effects than AT, with no significant statistical differences among the other blood components (Figure 4D).

### 3.5 Ranking of treatment effects for blood components

**Figure 5:**
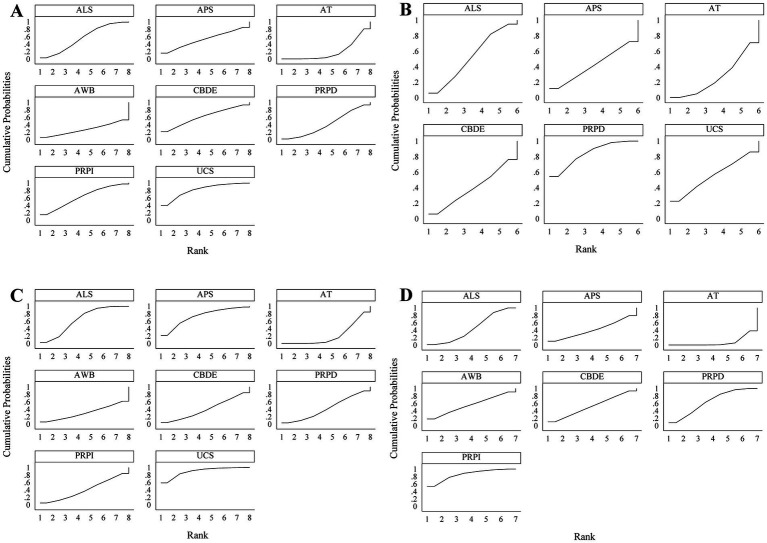
SUCRA results. **(A)** Schirmer’s I value; **(B)** CFSS; **(C)** TBUT; **(D)** OSDI. A larger area under the curve indicates better treatment efficacy of the respective blood component.

According to the SUCRA rankings, UCS demonstrated the best performance in improving Schirmer’s I value, followed by PRPI and CBDE; PRPD showed the most effective reduction in CFSS, followed by UCS and ALS; UCS was the most effective in improving TBUT, with APS and ALS following; PRPI exhibited the most significant reduction in OSDI, followed by PRPD and AWB (Figure 5).

### 3.6 Publication bias detection

**Figure 6:**
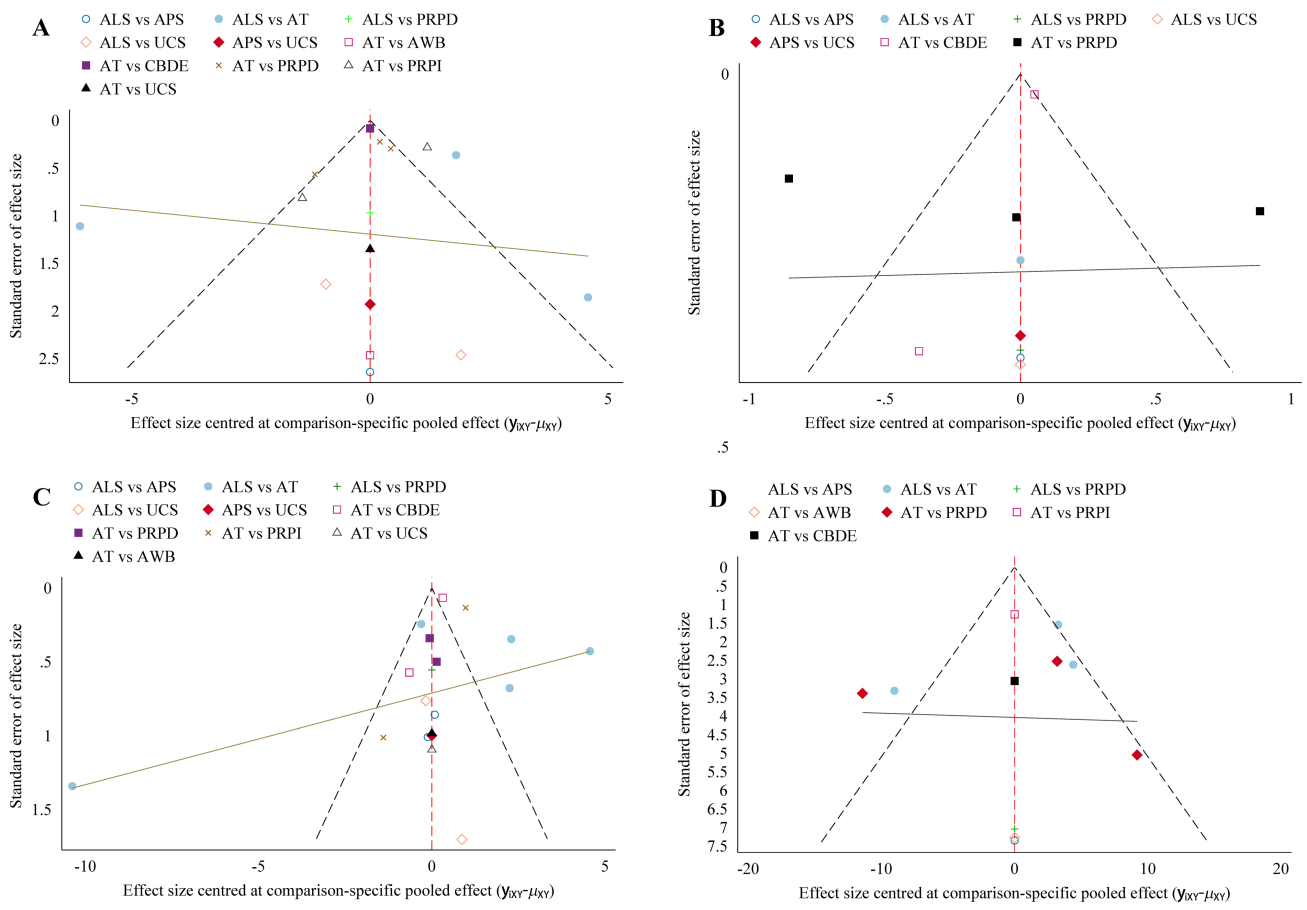
Comparison-adjusted funnel plot. **(A)** Schirmer’s I value; **(B)** CFSS; **(C)** TBUT; **(D)** OSDI. The more symmetrical the funnel plot, the lower the likelihood of publication bias and small sample effects.

Funnel plot analysis for each outcome measure indicated some asymmetry, suggesting the potential presence of publication bias and small sample effects (Figure 6).

## 4 Discussion

Dry eye disease is a prevalent chronic condition characterized by several key pathophysiological mechanisms, including tear film instability, increased tear osmolarity, ocular surface inflammation and damage, and abnormal neural function. In recent years, there has been a growing recognition of inflammation and hyperosmolarity as fundamental causes of dry eye disease, leading to a shift in treatment strategies from traditional AT to anti-inflammatory and immunomodulatory agents, as well as biological substitutes such as ALS and PRP (33, 34). This study comprehensively evaluates the efficacy of six blood components in the treatment of dry eye disease, analyzing them based on four clinical indicators. The results indicate significant differences in the effectiveness of various blood components in improving dry eye symptoms, reflecting the diversity of these components in ocular surface repair, anti-inflammatory effects, and enhancement of tear film stability.

Tear secretion and TBUT are important indicators for assessing the severity of dry eye disease and are commonly used in clinical trials to determine the extent of the condition. The stability of the tear film relies not only on the volume of tear secretion but also is closely correlated with the quality of the tears. Studies have shown that levels of inflammatory factors in the conjunctival epithelial cells and tear fluid of dry eye patients are significantly negatively correlated with tear film stability and tear secretion (35). This suggests that an increase in inflammatory factors exacerbates the inflammatory response on the ocular surface and contributes to tear film instability, thereby worsening dry eye symptoms. Anti-inflammatory factors present in ALS and UCS, such as TGF-*β* and IL-1RA, can effectively suppress chronic inflammation on the ocular surface and reduce the detrimental impact of inflammatory mediators on tear film stability. Furthermore, ALS and UCS contain nutrients similar to those found in natural tears, which help improve the ocular surface microenvironment and restore normal tear film function (36). Consequently, the effectiveness of ALS and UCS in improving TBUT is significantly superior to that of AT. Other blood components, such as PRP and APS, did not show significant effects on improving TBUT, suggesting potential differences in the mechanisms by which various blood components enhance tear film function. Yoon (37) compared the components of ALS and UCS, finding that the tear fluid component levels in UCS were higher than those in ALS. However, network meta-analysis based on Schirmer’s I value indicates no statistically significant differences among the various blood components in increasing tear secretion. Despite UCS and PRP being rich in growth factors and cytokines [such as platelet-derived growth factor (PDGF) and epidermal growth factor (EGF)], which may aid in repairing lacrimal gland structure and/or alleviating ocular surface inflammation, the restoration of tear secretion may be limited by structural damage to the lacrimal glands or the underlying pathological conditions present in the patients. Therefore, the direct effects of blood components may be somewhat constrained. As an emerging blood component, UCS exhibits stronger anti-inflammatory and immunomodulatory functions due to its unique constituents, such as placental growth factor and immune regulatory factors. Recent studies have shown that UCS can effectively reduce inflammation on the ocular surface of dry eye patients and enhance Schirmer’s I value and TBUT by promoting the regeneration of ocular surface cells (38). This is also reflected in the SUCRA analysis, which suggests that UCS may have the best therapeutic effect. This indicates that UCS not only plays a significant role in tear film stability but also demonstrates substantial potential in promoting tear secretion.

In the context of corneal epithelial injury repair, the results of a network meta-analysis based on CFSS indicate that the therapeutic effect of PRPD is significantly superior to that of AT. This difference may be attributed to the high concentration of growth factors present in PRP, particularly its ability to accelerate the proliferation and migration of corneal epithelial cells, thereby promoting the healing of damaged corneas (39). The platelet content in PRP is approximately 2.5 times that of whole blood, resulting in a richer supply of growth factors and other platelet-derived components (8). Previous studies have demonstrated that PDGF and EGF play critical roles in corneal epithelial repair, and the local application of PRP significantly increases the concentrations of these factors, enhancing the repair capacity of corneal tissue (40). Moreover, PRP exhibits anti-inflammatory properties, which can reduce the infiltration of inflammatory cells on the ocular surface, aiding in the alleviation of chronic damage and inflammatory responses (41, 42). This finding aligns with further SUCRA results, suggesting that PRP may have the best efficacy in improving corneal damage compared to ALS. This is likely due to PRP containing a greater abundance of growth factors and cell adhesion molecules essential for ocular surface healing compared to ALS (43). However, no significant differences were observed among other blood components in improving CFSS, indicating that the effectiveness of different blood components in corneal epithelial repair may depend on their formulation and individual patient characteristics. Blood components such as ALS and UCS are also rich in growth factors, but variations in their preparation methods, concentrations, and local release profiles may lead to different clinical outcomes. Compared to PRP, the concentrations of growth factors in ALS and UCS are relatively lower, which may necessitate longer treatment durations or higher application frequencies to achieve similar effects. Regarding the alleviation of subjective symptoms in patients, the results of a network meta-analysis based on the OSDI indicate that the therapeutic effects of ALS and PRP are significantly superior to those of AT. Additionally, no statistically significant differences were found among other blood components in improving patients’ OSDI scores. This is consistent with previous systematic reviews and meta-analyses. The meta-analysis by Akowuah et al. (11) demonstrated that PRP significantly improves subjective dry eye symptoms compared to AT. Conversely, PRP and ALS exhibited similar effects in alleviating dry eye symptoms. Both PRP and ALS contain growth factors that not only alleviate discomfort by providing lubrication and promoting ocular surface repair but may also improve long-term symptom control by reducing chronic inflammation on the ocular surface (44, 45). The improvement in OSDI reflects an enhancement in the overall quality of life for patients, indicating the multidimensional role of blood components in alleviating dry eye symptoms (46). However, due to the loss of platelets during the preparation and dilution of ALS, PRP is more enriched in growth factors compared to ALS, which explains why SUCRA predicts that PRP may have the best effect in improving OSDI.

Currently, several traditional meta-analyses have been published exploring the efficacy of blood component therapies for dry eye disease. However, this study represents the first network meta-analysis in this field, offering significant advantages over previous research. Wang et al.’s (12) meta-analysis, which was based on seven RCTs, compared the efficacy of ALS and AT in treating dry eye disease. The results indicated that ALS significantly improved the OSDI, TBUT, and CFSC, thereby alleviating dry eye symptoms compared to AT. Similarly, Quan et al. (13) analysis of six RCTs yielded comparable findings. While we incorporated a larger number of RCTs and also found that ALS significantly outperformed AT in improving dry eye symptoms, our results not only included data from direct comparisons but also strengthened the reliability of our findings through indirect comparisons. Therefore, our study provides more robust evidence in support of these conclusions. Additionally, a meta-analysis by Akowuah et al. (11) which included 10 observational studies and nine self-controlled studies, demonstrated that PRP can significantly alleviate the symptoms and signs of dry eye disease. This conclusion partially supports our findings; however, the credibility of our results, derived from RCT data, is notably higher. Furthermore, there are currently no additional meta-analyses investigating the efficacy of various blood components in treating dry eye disease. The existing meta-analyses primarily focus on the effects of ALS or PRP, leaving the therapeutic effects of other blood components unexamined. Our network meta-analysis consolidates data from six different blood components, providing a comprehensive and reliable source of evidence for current clinical practice in comparing their efficacy in treating dry eye disease.

The observed differences in clinical indicators among various blood components suggest that personalized treatment options can be tailored based on the specific pathological characteristics of patients. In patients with aqueous-deficient dry eye disease, UCS demonstrates significant advantages in enhancing Schirmer’s I values and TBUT, making UCS a preferred treatment option for this patient group. This choice is based on UCS’s effectiveness in improving tear secretion and stabilizing the tear film, which helps alleviate dry eye symptoms. Conversely, for patients with more severe corneal epithelial damage, PRP is more suitable due to its ability to promote epithelial repair. Rich in growth factors, PRP accelerates the regeneration and repair of corneal epithelial cells, making it a priority treatment choice for patients with corneal injuries in clinical settings. To implement these personalized treatment strategies, physicians must conduct a comprehensive assessment of patients’ tear secretion levels, tear film stability, and corneal health. Based on these evaluations, physicians can select the most appropriate blood component for treatment, thereby enhancing therapeutic outcomes and patient satisfaction. This differentiated treatment approach not only provides new insights for the precise management of dry eye disease but also underscores the importance of customizing treatment plans according to individual patient characteristics in clinical practice. Future research should further explore the potential applications of other blood components in different subtypes of dry eye disease to support broader personalized treatment strategies.

### 4.1 Advantages and limitations of this study

To our knowledge, this is the first network meta-analysis of RCTs evaluating the efficacy of blood components in the treatment of dry eye disease. The inclusion of high-quality RCTs establishes a solid foundation for the reliability of the meta-analysis results. We employed strict inclusion and exclusion criteria to ensure comparability among the studies included in the analysis. Additionally, we calculated the differences in outcome measures before and after treatment and utilized these in the network meta-analysis to ensure the accuracy of the results.

However, the limitations of this study must also be acknowledged. First, the asymmetrical funnel plot observed in this study suggests the potential for publication bias and small sample effects, particularly in some smaller RCTs, where exaggerated treatment effects or insufficient statistical power may impact the accuracy of the results. Therefore, future research should aim to increase sample sizes as much as possible to enhance the reliability of the findings. Second, the potential heterogeneity among different studies may reduce the reliability of the meta-analysis results. Variations in treatment frequency of blood components, the severity of dry eye disease among patients, age-related baseline characteristics, and differences in follow-up duration may introduce heterogeneity that affects the overall reliability of the meta-analysis. Consequently, future studies should standardize treatment protocols for blood components and meticulously document and report key information such as baseline characteristics of patients and follow-up durations. Moreover, some of the included studies lacked detailed reporting on randomization, allocation concealment, and blinding procedures, which may affect the overall quality of the research and the reliability of the results. Therefore, future studies should strengthen the implementation and detailed reporting of key methodological aspects such as randomization, allocation concealment, and blinding to improve the overall quality of the research and the reliability of the outcomes. Large-scale, multicenter RCTs are needed to further validate the efficacy of blood component therapies for dry eye disease. In fact, conducting subgroup analyses based on factors such as the severity of dry eye disease and patient age would provide valuable insights into the use of blood components in specific contexts. However, due to the mixed reporting of data from patients with varying severities of dry eye disease in the included studies, as well as the lack of clear reporting on the severity of dry eye disease in some studies, further subgroup analyses could not be performed. Additionally, the reporting details regarding age as a factor in the included studies were too vague, hindering the execution of subgroup analyses. Therefore, future research needs to provide more detailed reporting of patients’ baseline characteristics and trial specifics to offer more comprehensive information for the treatment of dry eye disease.

## 5 Conclusion

Blood components can significantly enhance tear secretion, improve tear film stability, and repair corneal epithelial damage in patients with dry eye disease, thereby markedly improving ocular surface health. Specifically, for patients with aqueous-deficient dry eye disease, UCS may represent the optimal treatment option, particularly due to its outstanding effects on enhancing tear secretion and stabilizing the tear film. In contrast, for patients with more severe corneal epithelial damage, PRP may offer a more effective treatment, as it has the ability to accelerate the regeneration and repair of corneal epithelial cells.

## Data Availability

The original contributions presented in the study are included in the article/Supplementary material, further inquiries can be directed to the corresponding author.
