# Supplementary material for: Blood component therapy for dry eye disease: a systematic review and network meta-analysis
Source: Front Med (Lausanne). 2024 Dec 16;11:1500160. doi: 10.3389/fmed.2024.1500160 (PMC11683103; doi:10.3389/fmed.2024.1500160)
Supplement: Supplementary file 1 [file Table_1.DOCX]

**Blood Component Therapy for Dry Eye Disease: A Systematic Review and Network Meta-Analysis**

**Table 1: Retrieval strategy**

| **1. PubMed**  #1: "Xerophthalmia"[MeSH Major Topic] OR "Dry Eye Syndromes"[MeSH Major Topic]  #2: ("xerophthalmia"[Title/Abstract] OR "dry eye"[Title/Abstract] OR "dry eye syndrome"[Title/Abstract] OR "corneal"[Title/Abstract]) AND "conjunctival xerosis"[Title/Abstract]  #3: #1 OR #2  #4: "Blood"[MeSH Terms] OR "Serum"[MeSH Terms] OR "Hemocytes"[MeSH Terms] OR "Blood Cells"[MeSH Terms] OR "Blood Platelets"[MeSH Terms] OR "Plasma"[MeSH Terms] OR "Erythrocytes"[MeSH Terms] OR "Leukocytes"[MeSH Terms]  #5: "blood"[Title/Abstract] OR "hematological"[Title/Abstract] OR "serum"[Title/Abstract] OR "blood serum"[Title/Abstract] OR "hemocyte"[Title/Abstract] OR "blood cell"[Title/Abstract] OR "blood cells"[Title/Abstract] OR "thrombocyte"[Title/Abstract] OR "platelet"[Title/Abstract] OR "platelets"[Title/Abstract] OR "plasma"[Title/Abstract] OR "plasmatic"[Title/Abstract] OR "erythrocyte"[Title/Abstract] OR "erythrocytes"[Title/Abstract] OR "red blood cell"[Title/Abstract] OR "erythrocytic"[Title/Abstract] OR "leucocyte"[Title/Abstract] OR "white blood cell"[Title/Abstract] OR "leukocyte"[Title/Abstract] OR "leukocytes"[Title/Abstract]  #6: #4 OR #5  #7: #3 AND #6  **2. WOS**  (TI=(blood OR hematological OR serum OR blood serum OR hemocyte OR blood cell OR blood cells OR thrombocyte OR platelet OR platelets OR plasma OR plasmatic OR erythrocyte OR erythrocytes OR red blood cell OR erythrocytic OR leucocyte OR white blood cell OR leukocyte OR leukocytes)) AND TS=(xerophthalmia OR dry eye OR dry eye syndrome OR corneal and conjunctival xerosis)  **3. Embase**  #1: (xerophthalmia:ab,ti OR 'dry eye':ab,ti OR 'dry eye syndrome':ab,ti OR corneal:ab,ti) AND 'conjunctival xerosis':ab,ti  #2: 'xerophthalmia'/exp  #3: 'dry eye'/exp  #4: 'dry eye syndrome'/exp  #5: #1 OR #2 OR #3 OR #4  #6: blood:ab,ti OR hematological:ab,ti OR serum:ab,ti OR 'blood serum':ab,ti OR hemocyte:ab,ti OR 'blood cell':ab,ti OR 'blood cells':ab,ti OR thrombocyte:ab,ti OR platelet:ab,ti OR platelets:ab,ti OR plasma:ab,ti OR plasmatic:ab,ti OR erythrocyte:ab,ti OR erythrocytes:ab,ti OR 'red blood cell':ab,ti OR erythrocytic:ab,ti OR leucocyte:ab,ti OR 'white blood cell':ab,ti OR leukocyte:ab,ti OR leukocytes:ab,ti  #7: 'blood'/exp  #8: 'serum'/exp  #9: 'blood serum'/exp  #10: 'hemocyte'/exp  #11: 'blood cell'/exp  #12: 'blood cells'/exp  #13: 'thrombocyte'/exp  #14: 'platelet'/exp  #15: 'plasma'/exp  #16: 'erythrocyte'/exp  #17: 'erythrocytes'/exp  #18: 'red blood cell'/exp  #19: 'leucocyte'/exp  #20: 'white blood cell'/exp  #21: 'leukocyte'/exp  #22: 'leukocytes'/exp  #23: #6 OR #7 OR #8 OR #9 OR #10 OR #11 OR #12 OR #13 OR #14 OR #15 OR #16 OR #17 OR #18 OR #19 OR #20 OR #21 OR #22  #24: #5 AND #23 AND [article]/lim AND [humans]/lim AND [clinical study]/lim  **4.Scopus**  TITLE-ABS-KEY ( "xerophthalmia" OR "dry eye" OR "dry eye syndrome" OR "corneal and conjunctival xerosis" ) AND TITLE-ABS-KEY ( "blood" OR "hematological" OR "serum" OR "blood serum" OR "hemocyte" OR "blood cell" OR "blood cells" OR "thrombocyte" OR "platelet" OR "platelets" OR "plasma" OR "plasmatic" OR "erythrocyte" OR "erythrocytes" OR "red blood cell" OR "erythrocytic" OR "leucocyte" OR "white blood cell" OR "leukocyte" OR "leukocytes" ) AND ( LIMIT-TO ( DOCTYPE , "ar" ) OR LIMIT-TO ( DOCTYPE , "re" ) )  **5. Cochrane**  #1: (xerophthalmia OR dry eye OR dry eye syndrome OR corneal and conjunctival xerosis):ti,ab,kw  #2: MeSH descriptor: [Xerophthalmia] explode all trees  #3: MeSH descriptor: [Dry Eye Syndromes] explode all trees  #4: MeSH descriptor: [Dry Eye Syndromes] explode all trees  #5: #1 or #2 or #3 or #4  #6: (blood OR hematological OR serum OR blood serum OR hemocyte OR blood cell OR blood cells OR thrombocyte OR platelet OR platelets OR plasma OR plasmatic OR erythrocyte OR erythrocytes OR red blood cell OR erythrocytic OR leucocyte OR white blood cell OR leukocyte OR leukocytes):ti,ab,kw  #7: MeSH descriptor: [Blood] explode all trees  #8: MeSH descriptor: [Hematology] explode all trees  #9: MeSH descriptor: [Serum] explode all trees  #10: MeSH descriptor: [Hemocytes] explode all trees  #11: MeSH descriptor: [Blood Cells] explode all trees  #12: MeSH descriptor: [Blood Platelets] explode all trees  #13: MeSH descriptor: [Plasma] explode all trees  #14: MeSH descriptor: [Erythrocytes] explode all trees  #15: MeSH descriptor: [Leukocytes] explode all trees  #16: #6 OR #7 OR #8 OR #9 OR #10 OR #11 OR #12 OR #13 OR #14 OR #15  #17: #5 AND #16 |
| --- |

**Table 2: Basic information of included studies**

| **No.** | **Author** | **Year** | **Country** | **Type of study** | **Degree of injury** | **Sample size** | **Age** | **Gender** | **Intervention** | **Treatment frequency** |
| --- | --- | --- | --- | --- | --- | --- | --- | --- | --- | --- |
| 1 | Mukhopadhyay | 2014 | India | RCT | Moderate to severe | 48 | / | / | UCS | 6 times a day for 6 weeks |
| 1 | Mukhopadhyay | 2014 | India | RCT | Moderate to severe | 52 | / | / | ALS | 6 times a day for 6 weeks |
| 1 | Mukhopadhyay | 2014 | India | RCT | Moderate to severe | 44 | / | / | AT | 6 times a day for 6 weeks |
| 2 | Noda | 2006 | Japan | RCT | / | 12 | 28.8±5.3 | / | ALS | 5 times a day for 6 months |
| 2 | Noda | 2006 | Japan | RCT | / | 15 | 31.2±6.2 | / | AT | 5 times a day for 6 months |
| 3 | Hassan | 2022 | UK | RCT | Moderate to severe | 25 | 64.07±9.95 | 4/26 | AWB | 4 times a day for 8 weeks |
| 3 | Hassan | 2022 | UK | RCT | Moderate to severe | 27 | 61.63±11.57 | 8/22 | AT | 4 times a day for 8 weeks |
| 4 | Celebi | 2014 | Turkey | RCT | Severe | 10 | 56.05±8.07 | 2/18 | ALS | 4 times a day for 4 weeks |
| 4 | Celebi | 2014 | Turkey | RCT | Severe | 10 | 56.05±8.07 |  | AT | 4 times a day for 4 weeks |
| 5 | Avila | 2018 | Colombia | RCT | Severe | 15 | 59.2±3.5 | 1/14 | PRPI | 5 times a day for 3 weeks |
| 5 | Avila | 2018 | Colombia | RCT | Severe | 15 | 52.7±3.5 | 1/14 | AT | 5 times a day for 3 weeks |
| 6 | Elessawy | 2021 | Egypt | RCT | Severe | 20 | 54±10 | 4/16 | PRPI | 3 injections in 2 months |
| 6 | Elessawy | 2021 | Egypt | RCT | Severe | 20 | 51±6 | 6/14 | AT | 3 injections in 2 months |
| 7 | García-Conca | 2018 | Spain | RCT | Moderate | 44 | 62.1±11.2 | / | PRPD | 6 times a day for 4 weeks |
| 7 | García-Conca | 2018 | Spain | RCT | Moderate | 39 | 66.2±11 | / | AT | 6 times a day for 4 weeks |
| 8 | Yılmaz | 2016 | Turkey | RCT | / | 24 | 25 ± 4.02 | 4/20 | ALS | 6 times a day for 8 weeks |
| 8 | Yılmaz | 2016 | Turkey | RCT | / | 24 |  | 4/20 | AT | 6 times a day for 8 weeks |
| 9 | Meer | 2021 | The Netherlands | RCT | Severe | 15 | 73±9 | 1/14 | ALS | 6 times a day for 4 weeks |
| 9 | Meer | 2021 | The Netherlands | RCT | Severe | 15 | 73±9 | 1/14 | APS | 6 times a day for 4 weeks |
| 10 | Berhuni | 2024 | Turkey | RCT | / | 33 | 54.04±7.94 | 34/96 | ALS | 6 times a day for 3 weeks |
| 10 | Berhuni | 2024 | Turkey | RCT | / | 32 |  |  | AT | 6 times a day for 3 weeks |
| 11 | Calvo | 2022 | Spain | RCT | Severe | 21 | 58.7±11.9 | 1/20 | ALS | 5 times a day for 12 weeks |
| 11 | Calvo | 2022 | Spain | RCT | Severe | 21 | 61.1±16.7 | 4/17 | APS | 5 times a day for 12 weeks |
| 11 | Calvo | 2022 | Spain | RCT | Severe | 21 | 63.8±7.8 | 3/18 | UCS | 5 times a day for 12 weeks |
| 12 | Kang | 2023 | Korea | RCT | / | 16 | 54.56±11.94 | 0/16 | ALS | 6 times a day for 12 weeks |
| 12 | Kang | 2023 | Korea | RCT | / | 14 | 54.07±11.28 | 0/14 | PRPD | 6 times a day for 12 weeks |
| 13 | Yue | 2023 | China | RCT | Moderate to severe | 45 | 53.82±7.33 | 18/27 | CBDE | 4 times a day for 4 weeks |
| 13 | Yue | 2023 | China | RCT | Moderate to severe | 45 | 52.96±7.68 | 17/28 | AT | 4 times a day for 4 weeks |
| 14 | Wu | 2021 | China | RCT | Moderate to severe | 31 | 35.74±0.08 | 11/20 | CBDE | 4 times a day for 4 weeks |
| 14 | Wu | 2021 | China | RCT | Moderate to severe | 22 | 36.86±7.75 | 8/14 | AT | 4 times a day for 4 weeks |
| 15 | Rawat | 2022 | India | RCT | Moderate to severe | 31 | 52.8 ± 12.8 | 11/20 | PRPD | 4-6 times a day for 12 weeks |
| 15 | Rawat | 2022 | India | RCT | Moderate to severe | 30 | 55.5 ± 13.4 | 12/18 | AT | 4-6 times a day for 12 weeks |
| 16 | Emam | 2021 | Egypt | RCT | Moderate to severe | 31 | 28-69 | 13/18 | PRPD | 4 times a day for 6 weeks |
| 16 | Emam | 2021 | Egypt | RCT | Moderate to severe | 31 | 27-72 | 11/20 | AT | 4 times a day for 6 weeks |
